# Relevance of Specific Surface of Mixed Oxide Derived from Layered Double Hydroxides on the Rheological Properties and Porosity of Cement Pastes

**DOI:** 10.1021/acsami.4c01949

**Published:** 2024-06-05

**Authors:** Caio C. dos Santos, Adriana A. Almeida, Sandra H. Pulcinelli, Celso V. Santilli

**Affiliations:** † Chemistry Institute of São Paulo State University (UNESP), Araraquara 14800-900, SP, Brazil

**Keywords:** LDH, Memory effect, Rheology control, Porosity control, Cement-based products, Portland cement

## Abstract

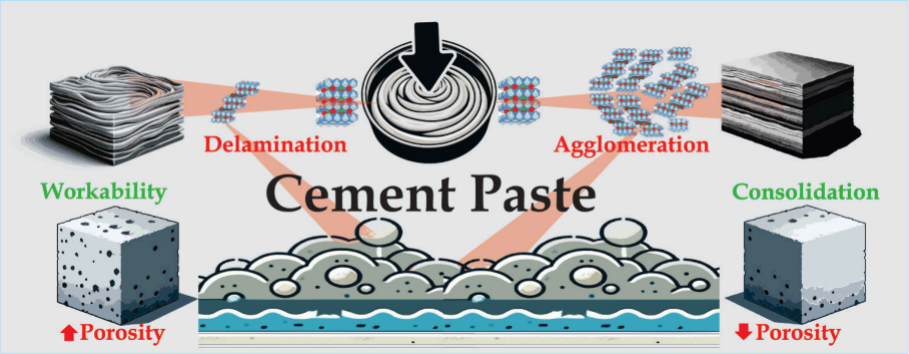

Cement-based products are the synthetic materials most used by humans, with consequent environmental impacts. One strategy that can assist in mitigating the adverse environmental effects of these materials involves the incorporation of multifunctional nanostructured additives. The objective of this work was to demonstrate the efficacy of incorporating mixed oxides (MO) derived from layered double hydroxides (LDH) to control the rheology and porosity of cement-based matrices. Thermal aging of LDH enabled the preparation of MO with different specific surface areas (SSA) for incorporation in different amounts in Portland cement. A low proportion of MO and low SSA increased workability by 22%. In contrast, a high proportion of MO and high SSA led to a 2.4-fold acceleration of cement consolidation and a 36.9% decrease of the porosity of the composite. These features could be attributed to additive–matrix interactions, with the LDH memory effect playing key roles in the cement crystal seed process and in competition for the absorption of free water within the cement paste. Therefore, the unprecedent results obtained suggest that the quantity and SSA of MO are key parameters to fine-tune the paste rheology and structure of hidrated cement. The MO materials showed easy adaptability and excellent potential for use as multifunctional additives in the production of eco-friendly, high-performance cement paste formulations with controllable properties according to the desired application.

## 1 Introduction

Increasing urbanization associated with population growth has focused attention on the environmental and social sustainability of large cities.^1−4^ This represents a major challenge that needs to be addressed by the sectors involved, especially those linked to the civil construction industry. Fulfilling the demands of society using the currently available construction technologies necessarily implies substantially increased pressure on the already threatened biosphere.^5−7^ Modern civil construction relies heavily on robust, low-cost, and easy-to-use mineral binders, with high availability from large-scale extraction of raw material.^8−13^ Cement-based products are the synthetic materials most used by man, with their worldwide production surpassing all other industries, including the areas of polymers and food.^14^

Cement production is associated with serious environmental problems, such as high anthropogenic CO_2_ emissions, which is of widespread concern due to the issue of climate change.^15^ It is estimated that more than 8% of anthropogenic CO_2_ derives from the production of calcium oxide (CaO) from raw materials based on calcium carbonate (CaCO_3_), according to eq 1. Estimates indicate that by 2050, these emissions could account for 30% of the total anthropogenic CO_2_ release.
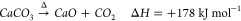
1

Hence, there is great urgency in identifying ways to reduce the environmental impacts associated with the cement industry, aiming at reducing greenhouse gas emissions and ensuring better living conditions for future generations.^16,17^ One promising approach is the development of new smart formulations containing multifunctional nanostructured additives that provide greater mechanical strength and durability, achieved by decreasing the porosity of cement-based composites, enabling the production of eco-efficient materials with superior mechanical-structural properties and high performance.^8,9,18^

Nanostructured cement additives can act as heterogeneous nucleating agents, accelerating the hydration processes and strengthening the interface transition zone between the paste and the aggregates (sand, stone, or lime) composing the concrete.^19^ The combination of such characteristics is highly attractive for applications in 3D additive manufacturing, where faster hardening of the paste is required. In addition, mechanical-structural improvements compatible with the prevention of corrosive processes in reinforced concrete can significantly increase the durability of civil structures.^20^

The layered double hydroxides (LDH) are potential candidates for applications as smart multifunctional additives in cement, enhancing performance.^21−24^ LDH are anionic clays with a structure represented by [M_*1-x*_^2+^ M_*x*_^3+^ (OH)_2_]^*x*+^ A_*x*/*n*_^*n*-^·zH_2_O, where M^2+^ and M^3+^ are metallic cations that occupy octahedral interstices of the layers, and A^n-^ represents the organic or inorganic anions present in the layer galleries. LDH are easily synthesized in a diversity of compositions at a low production cost, which, coupled with their unique properties, make these materials promising for applications in different areas. Specifically in the case of cementitious materials, the incorporation of LDH can be an attractive strategy for rheological control of the cement paste, in addition to providing improvements in terms of hardness, corrosive ion scavenging, and chemical stability.^25−27^

LDH undergoes thermal decomposition at moderate temperatures, resulting in the formation of nanocrystalline mixed oxides (MO) that can recover the LDH structure by means of a memory effect, when in contact with water or anionic solutions.^6^ The memory effect of LDH, coupled with its anion exchange capacity, can promote the filling of tiny pores and the sequestration of undesirable anions, such as carbonate and chloride. Furthermore, the high specific surface area (SSA) of nanostructured MO compared to cementitious phases favors the heterogeneous nucleation of cement hydration products. Therefore, all these unique properties of LDH will directly impact the structural and chemical characteristics of cementitious materials.^21,28^

Few works have explored the potential of using the LDH memory effect to reduce the porosity and control the rheological and chemical characteristics of cement.^25^ Once incorporated, the MO can undergo dissolution and reprecipitation reactions during the cement hydration process.^29^ The Ostwald–Freundlich model predicts that under chemical equilibrium conditions, precipitation preferentially occurs in nanometric pores of the solid matrix.^29,30^ Furthermore, few studies have reported on the association between the LDH memory effect and the rheology of the cement paste. The MO reactions can influence the structuring kinetics of the paste by accelerating the increase of the solid quantity reacting with water, due to the changes in morphology and the competition for water capture.^31^ Finally, so far, there appear to have been no studies exploring correlations between the SSA of MO additives and the processing and structural features of cement.

This work evaluated the effects of the mass fraction and SSA of MO cement additives derived from LDH with intercalated nitrate ions (Mg_0.66_Al_0.34_(OH)_2_(NO_3_)_0.17_·zH_2_O). Thermal aging of LDH was used to prepare MO additives with different SSA. The MO was incorporated in the cement paste to modify the rheology and reduce the porosity by means of the LDH memory effect. In terms of rheology, additive mass fractions of 1% and 3% acted to decrease and increase the paste viscosity, respectively. The elastic modulus was highly sensitive to additive SSA, with an increase of up to 2.4-fold using a 3% mass fraction of the MO with the highest SSA. Cement porosity showed linear and parabolic relationships with MO quantity and SSA, respectively, indicating that high SSA enhanced filling of the cement matrix pores by additive interaction. The results suggested that the LDH memory effect competed with cement paste water, enabling control of the final properties of the cementitious material.

## 2 Experimental Section

### 2.1 Materials

All the chemicals used in the synthesis and characterization of the samples were of analytical grade. The ultrapure water used in all the steps was heated to eliminate possible dissolved gases and ensure that carbonate-type contaminants were absent. Sodium hydroxide (NaOH, 97%, CAS 1310–73–2), magnesium nitrate (Mg(NO_3_)_2_·6H_2_O, 98%, CAS 1344–18–9), isopropyl alcohol (C_3_H_8_O, 99.5%, CAS 67–63–0), and ethyl ether ((C_2_H_5_)_2_O, 98%, CAS 60–29–7) were obtained from Exôdo Científica. Aluminum nitrate (Al(NO_3_)_3_·9H_2_O, 98%, CAS 7784–27–2) and sodium nitrate (NaNO_3_, 99%, CAS 7631–99–4) were purchased from Neon. These chemicals were used as received. High early strength Portland cement (CP V), manufactured by Holcim (São Paulo, Brazil), was used in this study. The cement presented a density of 3.1 g·mL^–1^ and a specific surface area of 1.5 m^2^·g^–1^.

### 2.2 Preparation of Samples

The synthesis of Mg_0.66_Al_0.34_(OH)_2_(NO_3_)_0.17_·zH_2_O LDH was carried out using the coprecipitation method at constant pH and a controlled temperature of 25 °C, following a well-known methodology.^32^ First, 72 mmol of Mg(NO_3_)_2_·6H_2_O and 48 mmol of Al(NO_3_)_3_·9H_2_O were dissolved in 250 mL of distilled water. This solution was then added dropwise to 1 L of a solution containing 72 mmol of sodium nitrate (NaNO_3_), with the pH adjusted to 10.0 by addition of 2.0 mol·L^–1^ sodium hydroxide (NaOH). The syntheses were performed in an inert atmosphere, with nitrogen (N_2_) as a purge gas. In order to obtain nanostructures with different specific surface areas, portions of the solution were aged at 60 °C for 0, 10, 20, and 30 h, resulting in the materials denoted LDH A, LDH B, LDH C, and LDH D, respectively. The precipitate (LDH) was centrifuged and washed repeatedly with ultrapure water to remove excess sodium ions. The LDH was frozen, lyophilized, macerated, passed through a sieve (125 mesh), and calcined at 500 °C for 1 h, using a heating rate of 10 °C·min^–1^, to obtain the MO.

The memory effect was evaluated using three different cement environments. Simulated cement pore solutions, denoted SCPS1 and SCPS2, were prepared at pH 14 and 8, respectively, by adding different salts, as shown in Table S1. Cement water (CW) at pH 12.5 was obtained from the supernatant after centrifuging a 1:1 (w/w) cement/water mixture at 8000 rpm for 5 min.

### 2.3 Cement Pastes

The MO was incorporated into the cement at different mass fractions (1, 2, and 3 wt %), followed by adding water at a constant water-to-cement weight ratio of 0.4 wt %. After water addition, the pastes were manually mixed for 30 s, followed by mechanical mixing for 3 min at 10,000 rpm, using a Makita RT0700C mixer, to ensure homogeneity of the samples, which were later used in the rheological assessments. The pastes were also used for molding cubic (10 mm^3^) test bodies that were placed in a controlled environment at 25 °C and 100% relative humidity, for different times (3, 7, 14, and 28 days). After these periods, the hydration reactions were interrupted using the solvent exchange method,^33^ where the samples were immersed in isopropanol and ethyl ether to replace the structural hydration water. Two cycles of immersion for 1 h in isopropanol (10:1 solvent/paste volume ratio) were followed by a third cycle with immersion for 24 h using a 50:1 solvent/paste volume ratio. In all these steps, isopropanol was used at 4 °C to minimize damage due to ettringite crystallization.^34^ A fourth cycle was added, with immersion of the samples in ethyl ether for 24 h, using a 15:1 solvent/paste volume ratio, followed by drying at 40 °C for 30 min.^33^

### 2.4 Characterization

#### 2.4.1 Powders

The crystalline phases were identified by powder X-ray diffraction (XRD) analysis, at room temperature, using a Rigaku SmartLab SE diffractometer operating with Cu Kα radiation (λ = 1.5418 Å), at 40 kV and 30 mA. The diffraction peaks were indexed and Rietveld refinement was performed using *SmartLab Studio II* software and Crystallography Open Database (COD) or Inorganic Crystal Structure Database (ICSD) files. The powder density was determined by helium pycnometry, using an AccuPyc 1330 instrument (Micromeritics).

Fourier transform infrared (FTIR) spectroscopy measurements employed a PerkinElmer Frontier FTIR spectrometer equipped with a diffuse reflectance infrared accessory (DRIFTS). The spectra were acquired at 4 cm^–1^ resolution, in the range from 4000 to 400 cm^–1^, with accumulation of 256 scans.

Thermogravimetric analysis (TGA) employed a TA Instruments SDT Q600 system, with the sample heated from 25 to 1000 °C, at 10 °C·min^–1^, under an atmosphere of nitrogen supplied at a flow rate of 100 mL·min^–1^.

The specific surface areas (SSA) of the LDH and MO powders, as well as the cement composite test specimens, were investigated using nitrogen adsorption–desorption isotherms, employing a Micromeritics ASAP 2010 analyzer. The samples were degassed for 12 h, under reduced pressure, at 200 and 40 °C for the additives (LDH and MO) and the cement composites, respectively. The analyses were performed at liquid nitrogen temperature, in the relative pressure range from 0.002 to 0.998, with determination of SSA according to the Brunauer–Emmett–Teller (BET) method.^35^

The micrograph of LDH samples was captured in a scaning electron microscope (SEM) JEOL JSM-IT500HR using a secondary electrons (SE) detector.

The surface charge and particle size distribution of the LDH samples were evaluated through the analyses of the zeta potential (ζ) and dynamical light scattering (DLS) using a Zetasizer Nano series ZS equipment from Malvern Instruments. The average hydrodynamic diameter was determined by cumulative analysis, with the final value being the average of the midre size triplicate. The Zeta potential was accessed by dispersing LDH powder in 1 mL of water solution (sodium nitrate 1 mmol L^–1^), followed by pH titration in the pH range 4.5 to 10 in triplicate with 30 s between each measurement, using nitrate acid and sodium hydroxide water solutions (0.1 mol L^–1^).

#### 2.4.2 Pastes

The rheological behaviors of the pastes were evaluated using a rotational rheometer (AR200 EX, TA Instruments) with parallel plate geometry (40 mm diameter and gap of 1000 μm) and Peltier-controlled plate temperature of 25 °C. A textured adhesive tape (Safety-walk, 3M) was attached to the surfaces of the plates to prevent the cement paste from sliding. Rotational and oscillatory tests were performed for three different batches of each mixture, with the measurement made 10 min after cement-water contact and subsequent mixing. For removal of the stress related to the sample preparation procedure and to reach the constant temperature condition, a preshear at 50 s^–1^ was applied for 1 min, followed by a rest for 2 min, before the flow and oscillatory tests.

The flow tests were performed in controlled shear rate mode, consisting of an acceleration (0–100 s^–1^) step of 2 min and a deceleration (100–0 s^–1^) step of 2 min. Dynamic yield stress (DYS) and plastic viscosity (PV) measurements enabled analysis of the fluidity of the paste and microstructural damage.^36,37^ Cement pastes show pseudoplastic fluid behavior that is in accordance with the Bingham model.^38^ The DYS and PV values were obtained from linear fitting of the Bingham plastic fluid model, represented by eq 2.
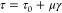
2where, τ (Pa), τ_0_ (Pa), μ (Pa·s), and γ (s^–1^) are the shear stress, DYS, PV, and shear rate, respectively.

Paste consolidation during hydration was monitored continuously for 4 h by oscillatory tests performed within the linear viscoelastic domain (LVED). To avoid a destructive process during consolidation, the oscillatory test was conducted at a constant frequency of 1 Hz and low strain amplitude of 10^–4^ rad.

#### 2.4.3 Porous Structure

The effects of the MO additives on the porous structures of the molded cement composites were evaluated at different times up to the 28^th^ day of aging after hydration. The porosity and pore size distribution were determined by mercury intrusion porosimetry, using an AUTOPORE III instrument (Micromeritics), in the pressure range between 0 and 414 MPa, corresponding to a pore size range between 0.003 and 360 μm. Before analysis, all the samples were degassed under vacuum, at pressure below 50 μPa.^25^ The pore size was calculated from the applied pressure values using the Washburn equation, with surface tension and mercury contact angle values of 0.489 N m^–1^ and 135°, respectively.^39^

#### 2.4.4 Regression Models

The measurements were performed in triplicate, using a new sample for each test. Multivariate regression models were constructed to assess the influence of the mass percentage and SSA of the additives. The data were processed and analyzed using *R* software and the *R studio* interface, with the *ExpAnalysis3d v. 0.1.1* and *Pacman v. 0.5.1* statistical packages for data processing.

## 3 Results and Discussion

### 3.1 LDH, MO, and LDHR

**Figure 1:**
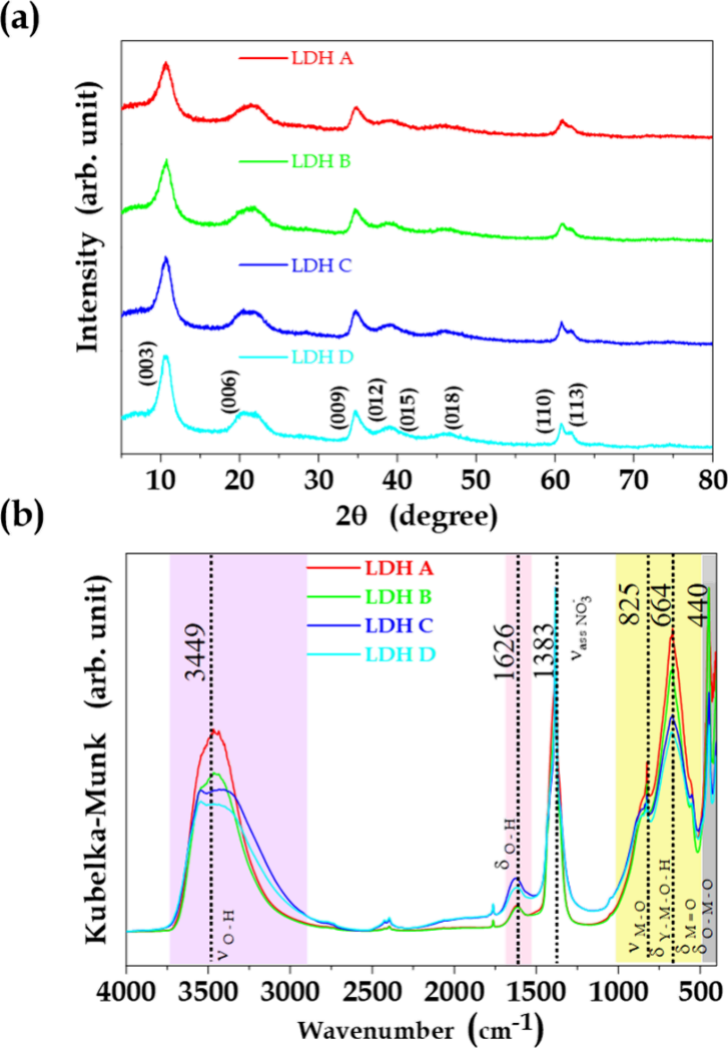
X-ray diffraction patterns (a) and FTIR spectra (b) of LDH samples aged at 60 °C during 0, 10, 20, and 30 h (LDH A to LDH D).

X-ray diffraction analysis was used to evaluate the effect of isothermal aging at 60 °C on crystallization of the LDH. The diffractograms (Figure 1(a)) showed the basal reflections typical of the lamellar structure, as evidenced by the (00*l*) peaks [(003), (006), and (009)], confirming formation of the Mg_0.66_Al_0.34_(OH)_2_(NO_3_)_0.17_·zH_2_O structure (JCPDS 14–0191).^40^ The basal spacing determined using the Bragg equation (d_00*l*_=nλ/2sin(θ)) was 0.83–0.84 nm, confirming the intercalation of nitrate ions in the interlamellar galleries of the synthesized LDH.^41,42^ Assuming 3R stacking of the layers, the average crystallite sizes determined using the Scherrer equation^43−45^ for the samples aged for 0, 10, 20, and 30 h were 3.05, 3.24, 3.68, and 4.07 nm, respectively, for the *c* direction (003), and 7.34, 8.30, 9.09, and 9.30 nm, respectively, for the basal *ab* plane direction (110). As shown in Table 1, irrespective of the aging time, the average crystallite sizes were 2.3–2.6 times larger in the *ab* plane direction than in the *c* direction. These results showed that increasing the aging time led to the formation of larger crystallites by the growth of lamellae and the aggregative stacking of layers in the *c* direction. The growth of lamellae under chemical equilibrium conditions is expected to occur due to the Ostwald ripening process, which is limited by solute diffusion.^46−48^ It should be noted that formation of the LDH nanostructure involves two main stages, namely nucleation and growth, which affect the size distribution of the crystallites. During coprecipitation at low supersaturation, small nuclei are formed in the first stage, followed by crystallite growth, oriented lamellae attachment (aggregation), and Ostwald ripening. The time invariance of the shape factor (CS_*ab*_/CS_*c*_ = 2.3–2.6) and the equal C/D ratio values determined for CS_*ab*_ and CS_*c*_ indicated that aggregative attachment and Ostwald ripening contributed similarly to anisotropic growth of the LDH crystallites.^46−48^

**Table 1:** Effect of Aging Time on SSA, Crystallite Size (CS), and Average Hydrodynamic Diameter Size by DLS of the LDH and MO Samples

SAMPLE	LDH A	LDH B	LDH C	LDH D	A/D ratio
SSA (m^2^·g^–1^)	88	73	16	5	17.1
CS*c* (nm)	3.1	3.2	3.7	4.1	0.8
CS*ab* (nm)	7.3	8.3	9.1	9.3	0.8
CS*ab*/CS*c*	2.4	2.6	2.5	2.3	1.0
DLS (nm)	169	202	203	330	0.5

SAMPLE	MO A	MO B	MO C	MO D	A/D ratio
SSA (m^2^·g^–1^)	220	176	98	54	4.1
CS*a* MgO (nm)	3.2	3.2	3.2	2.9	1.1
CS*ab*_LDH_/CS*a*_MO_	2.3	2.6	2.8	3.2	
SSA_MO_/SSA_LDH_	2.5	2.4	6.0	10.5	0.2

The platelet shape of the LDH primary particles was confirmed from SEM images displayed at Figure S1, that shows the well-known sand rose aggregated structure. The comparison of LDH A, and LDH D images evidence the platelet preservation during the aging periods, which supports the hypothesis of anisotropic growth of LDH. More reprentative information on the aggregation growth was obtainet from the average hydrodynamic diameter size of agragates present in the dispersion at pH 10 (Table 1), as evaluated from dynamic light scattering (DLS). The observed agglomerates growth during LDH aging display an A/D ratio similar to that of crystallite growth. Finaly the effect of aging on the surface properties of LDH were analised from zeta potential titration of samples in the pH range 4.5 to 10. Insignicant zeta potential change (35–38 mV) was observed inrrespective of LDH aging and pH. These findings suggest that, given the similar surface properties and particles morphology of different LDH, the primary factor to be considered in the additive properties is the modification of the specific surface area (SSA) caused by the growth of platelets during aging.

The infrared spectra showed vibration bands consistent with the intercalation of nitrate anions in the LDH (Figure 1(b)). A broad band centered at 3465 cm^–1^ could be attributed to the stretching of surface hydroxyl groups in the lamellae and water molecules, in agreement with a weak band at 1610 cm^–1^ attributed to deformation of the H_2_O molecule. An intense characteristic band at 1383 cm^–1^ was attributed to asymmetric stretching of nitrate.^49^ A broad band at 664 cm^–1^, together with a narrow and intense band at 440 cm^–1^, could be attributed to the stretching and deformation modes of the metallic oxides (M-O).^49,50^ Aging of the LDH affected the region between 1000 and 500 cm^–1^, containing the bands attributed to M-O and M–O-H, as well as absorptions of Y-M–O-H species (where Y refers to Al = O), which were more prevalent in surface regions.^49,50^ Decreases of the intensities of these bands reflected the LDH growth mechanism. In addition, the SSA determined from N_2_ adsorption isotherms also decreased significantly with aging, due to anisotropic crystallite growth and the attainment of fully dispersed lamellae (Table 1).

**Figure 2:**
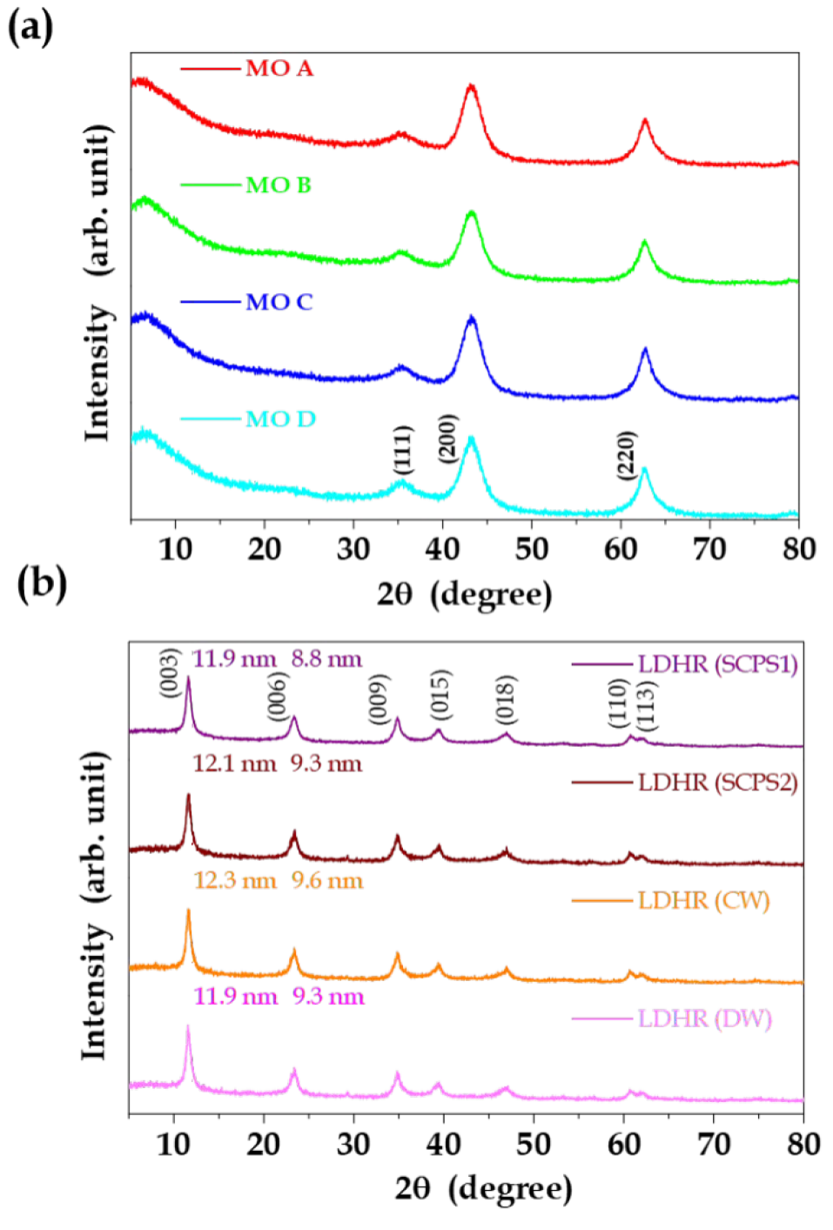
(a) X-ray diffractograms of the MO resulting from calcination of LDH A, LDH B, LDH C, and LDH D. (b) X-ray diffractograms of the LDHR regenerated from MO derived from LDH A in simulated cement and concrete pore solutions (SCPS1 and SCPS2), cement water (CW), and deionized water (DW). The crystallite sizes calculated using the *c* and *ab* plane directions are shown at left and right, respectively.

The X-ray diffractograms of the MO obtained from calcination of the different LDH showed diffraction peaks characteristic of MgO (Figure 2(a)). The collapse of the LDH structure was evidenced by the absence of the characteristic lamellar peaks. Surprisingly, the MgO crystallite size, calculated from the *a* plane reflection and the MO SSA, decreased as the LDH aging time increased. This antagonistic behavior indicated that the MO SSA values included a minor contribution from MgO crystallites and a dominant contribution from the amorphous phase (Table 1). Comparison of CS*ab*_LDH_ and CS*a*_MO_ evidenced that for the samples with short aging times (LDH A and LDH B), dehydroxylation of the LDH caused 2.3–2.6-fold reductions of crystallite size and similar magnitude increases of SSA. In the case of the samples with long aging times (LDH C and LDH D), the expected square root relation between increase of SSA and decrease of the crystallite size was observed. These findings confirmed the contribution of the amorphous MO phase to the substantial increase of SSA after calcination of the LDH precursors with the highest initial SSA (LDH A and LDH B).

The exposure of the MO derived from LDH A to different solutions led to the regeneration of layered double hydroxide (LDHR) by the memory effect. Figure 2(b) shows X-ray diffractograms of the samples regenerated in simulated cement and concrete pore solutions (SCPS1 and SCPS2), cement water (CW), and deionized water (DW). The diffractograms indicated recovery of the LDH structure after 24 h in contact with the simulated solutions. The calculated crystallite sizes were very similar for use of the different solutions, with sizes of approximately 12 and 9.3 nm in the *c* and *ab* directions, respectively. The interlamellar distances were 0.77 nm, demonstrating that for all the regenerations, the LDH was intercalated with carbonate anions, confirming the ability of the additive to scavenge this anion considered harmful to the metallic structures of reinforced concrete.^23,51^

These results highlighted that MO derived from LDH calcination could promote increases of SSA and reactivity of the cement precursors, with recapture of carbonates from the hydration medium and regeneration of the lamellar structure. The findings demonstrated the potential of MO as an eco-efficient cementitious additive with advanced properties. As discussed in the following section, the memory effect and high SSA of LDH can be used to induce the heterogeneous nucleation of hydrated cement phases, since the values obtained are much higher (∼200 m^2^g^–1^) than that observed for cement (15 m^2^g^–1^), affecting the paste rheology and the final structure.^25^

### 3.2 Rheological Properties of the Cement Paste with Added MO

#### 3.2.1 Flow Behavior

**Figure 3:**
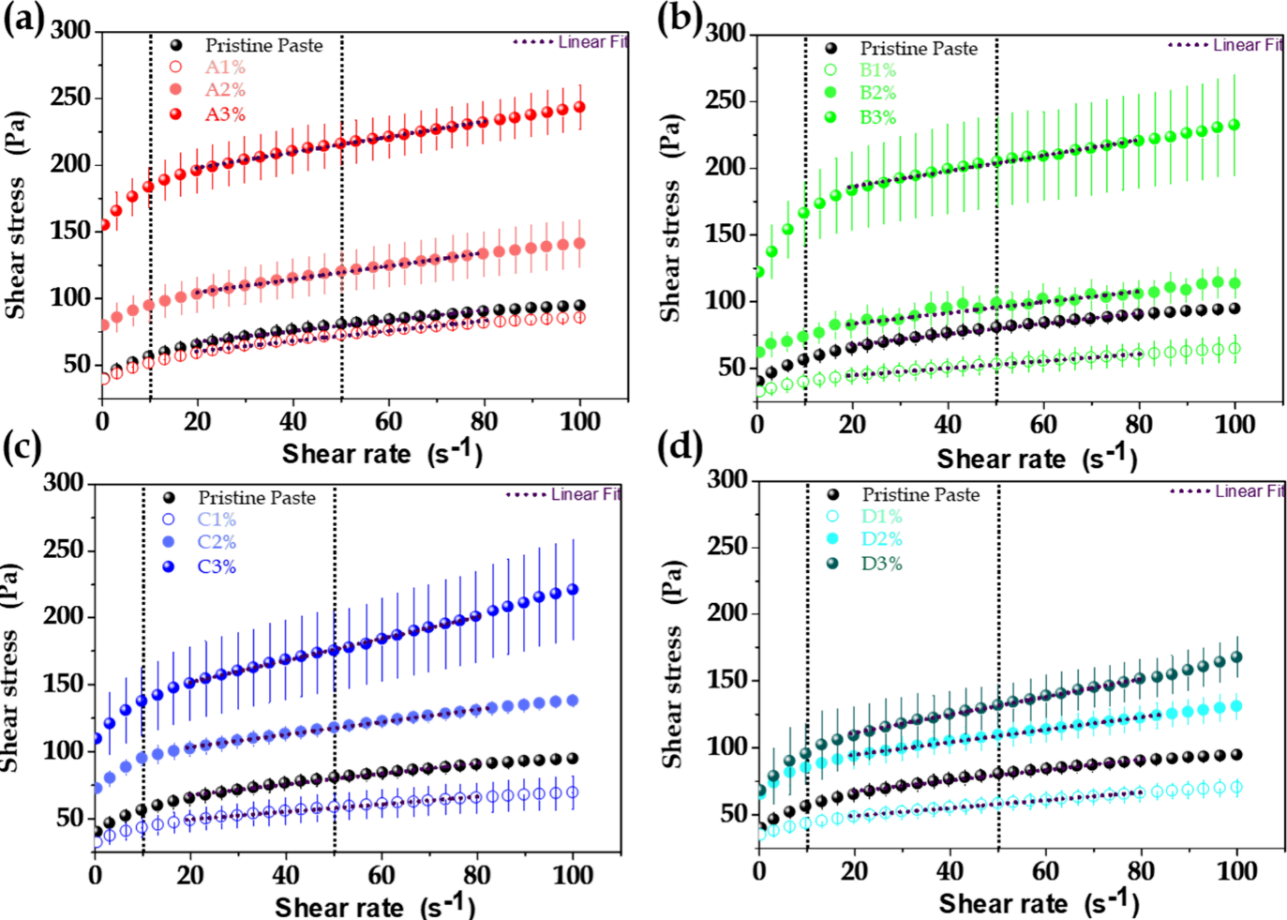
Shear stress as a function of shear rate for cement formulations containing different amounts of MO (0, 1, 2, and 3 wt %) with controlled SSA: (a) MO A, 220 m^2^·g^–1^, (b) MO B, 176 m^2^·g^–1^, (c) MO C, 98 m^2^·g^–1^, and (d) MO D, 53 m^2^·g^–1^. The vertical dashed lines at 10 and 50 s^–1^ indicate the shear rates used to derive the response surfaces.

The rheology of hydrated cement paste has implications for its application, consolidation, durability, strength, and workability.^38,52,53^ The effects of the quantity and SSA of MO incorporated in the cement paste formulations were first evaluated using the downward flow curves determined 10 min after contact with water (Figure 3). The flow curves were used to fit the Bingham model over the entire shear rate interval, with coefficient of determination (R^2^) values ranging from 0.950 to 0.998. For all the curves, the points at low shear rate (0–15 s^–1^) were slightly below the linear fitting, indicating a small shear thinning effect.

Irrespective of the MO sample, the additive content directly affected the flow properties of the paste. For the lowest MO addition (1 wt %), the shear stress decreased, compared to the pristine cement paste. This effect was more significant for the paste prepared with MO B, characterized by an intermediate SSA (176 m^2^·g^–1^). Increases of shear stress were observed for the samples with 2 and 3 wt % MO addition, which could be attributed to adsorption of free water by the MO, together with the formation of open agglomerates of particles. Irrespective of the SSA, the addition of a small amount of MO (1 wt %) led to easy fluidity of the cement paste, which could be explained by the lubricating effect of delaminated LDH platelets.^25^ The results showed that the SSA of MO significantly contributed to the paste shear stress, which increased proportionally with the SSA of the additive.

**Figure 4:**
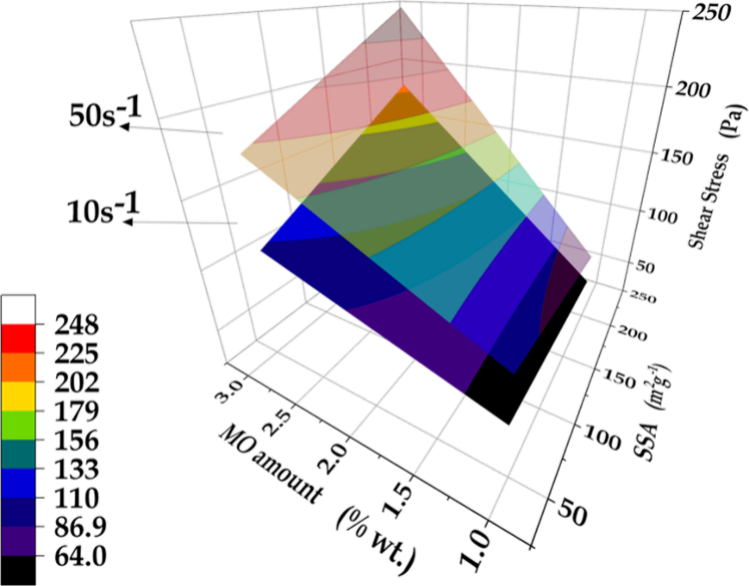
Response surfaces for shear stress, with two shear rate values (10 and 50 s^–1^), as a function of the SSA and amount of MO added to the cement paste.

The flow curve was used to construct response surface models (Figure 4), considering the shear stress (*Ss*) values corresponding to shear rates of 10 and 50 s^–1^ (vertical dashed lines in Figure 3). The regression models obtained considering the minimal terms for *Ss* of 10 and 50 s^–1^ are given by eqs 3 and 4, respectively:
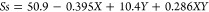
3
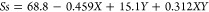
4where, X and Y are the SSA and quantity of MO, respectively.

Comparison of the first order coefficients showed that the contribution of the MO quantity was almost thirty-fold more important for increasing the shear stress. The contribution of the XY interaction (interaction between SSA and quantity of MO) was very small and of the same order of magnitude as the first order coefficient for X (representing SSA). The response surface was almost plane, while the small dependence of X, Y, and XY on increase of the shear stress from 10 to 50 s^–1^ was a consequence of the small shear thinning contribution to the deviation of the plastic behavior. The considerable difference in the magnitudes of the X and Y coefficients highlighted that in the initial step of the cement hydration, the MO quantity played a more significant role than the SSA and was the primary parameter responsible for the substantial alteration of the shear stress of the pastes.

The dependence of the dynamic yield stress (DYS) and plastic viscosity (PV) on the SSA and amount of MO in the cement pastes is shown in Figures S2 and S3, respectively. As expected from the flow curves, the lowest DYS and PV values (lower than for the pristine paste) were obtained for the samples with 1 wt % of MO, irrespective of the SSA values. The slight reductions of DYS and PV, relative to the pristine paste, suggested that with vigorous agitation during the synthesis, there was rapid hydration of the additive in the cement paste, and that only a small amount of water was consumed in this process. In addition, rapid hydration of the MO would lead to formation of the lamellar structure of the LDH, which could favor a laminar flow of the paste in the direction of shear stress, decreasing DYS and PV. When the quantity of MO additive was increased, there was greater competition between the paste and the additive for water consumption, which became more pronounced as the MO SSA increased. Furthermore, a significant amount of MO would induce stacking of the layers and the consequent growth of LDH aggregates, resulting in higher values of DYS and PV.

**Figure 5:**
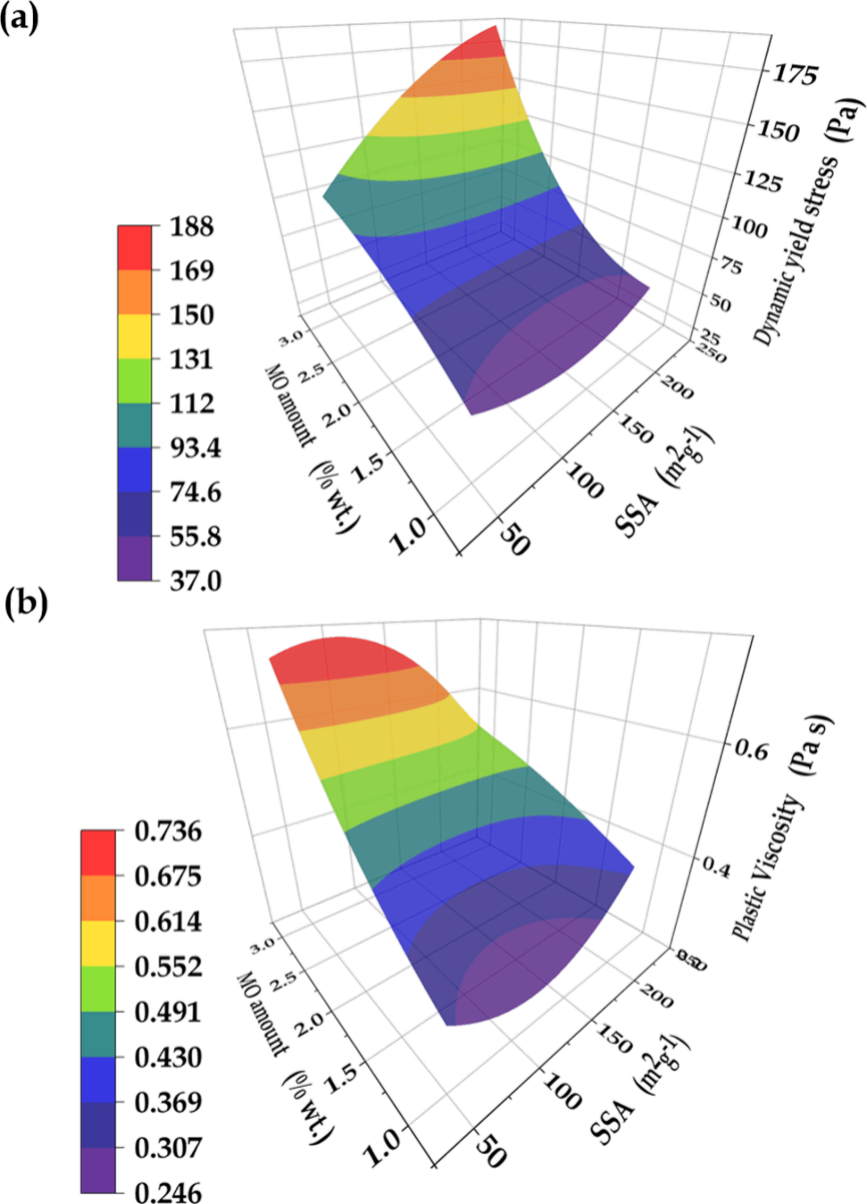
Response surfaces for (a) dynamic yield stress (DYS) and (b) plastic viscosity (PV) of the cement paste, as a function of MO amount and SSA.

Figure 5 shows the response surfaces for DYS and PV, obtained using the models described by polynomial eqs 5 and 6, where X and Y are the MO SSA and the MO quantity, respectively. Comparing the first order coefficients in eq 5, the amount of MO additive had an almost 20-fold greater impact on DYS, compared to the effect of SSA. In addition, comparison of the three second order coefficients revealed a small contribution of SSA to the curvature of the response surface. Accordingly, DYS increased almost linearly with SSA, while a pronounced parabolic increase occurred with increasing amount of higher SSA MO. This behavior evidenced an important contribution of the interaction between the parameters MO amount and SSA to the increase of DYS. It can also be seen from eq 6 that the MO amount had an impact on PV that was 20 times greater than that of SSA. These observations confirmed that the MO amount was the main parameter affecting the flow properties. This was probably due to competition between the MO additive and the cement for initial water consumption, as well as the heterogeneous nucleation and growth of open aggregates of cement phases, induced by regenerated LDH platelets.
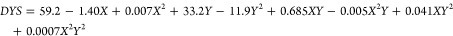
5
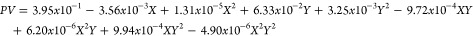
6

#### 3.2.2 Oscillatory Behavior

**Figure 6:**
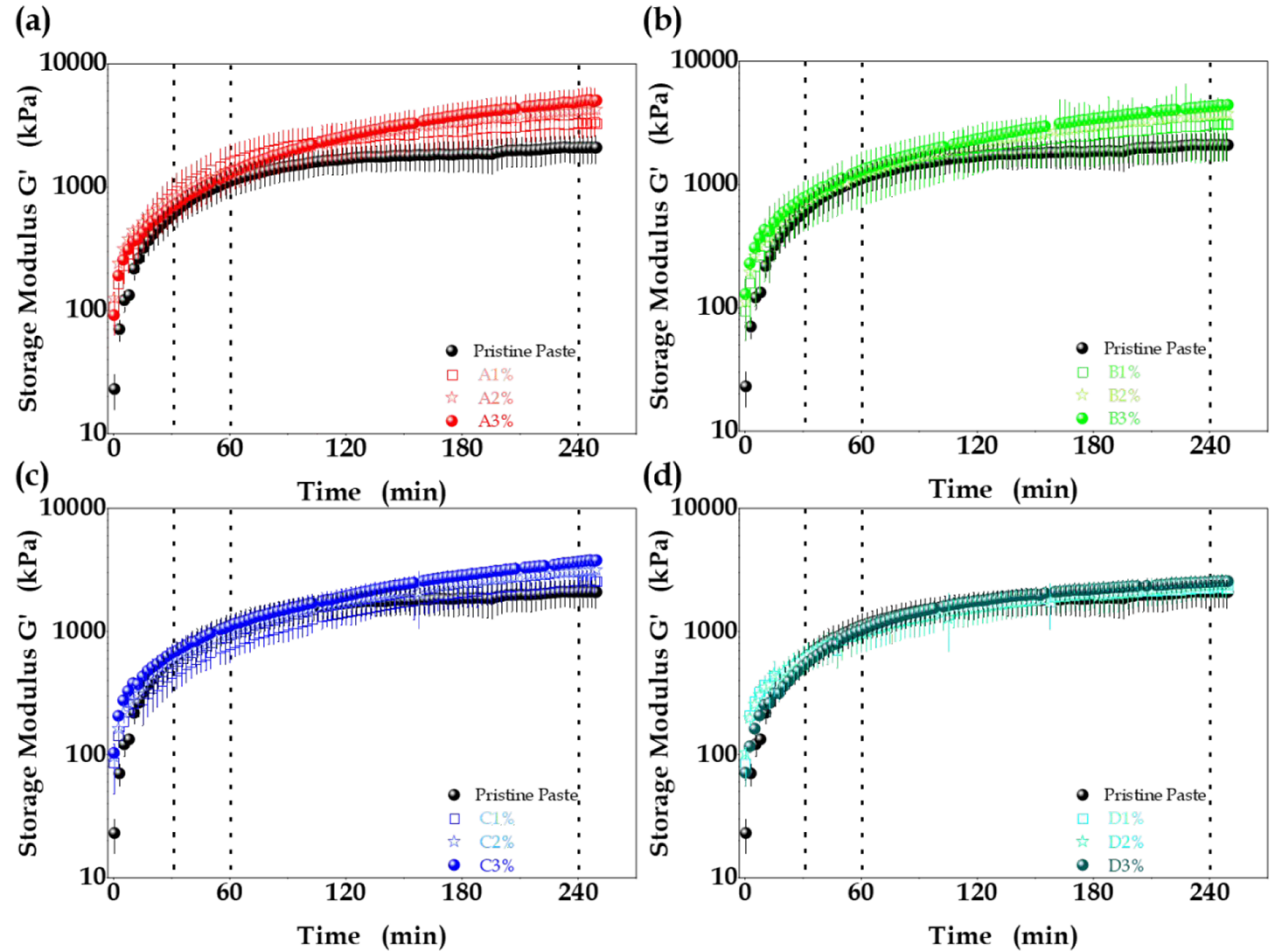
Storage modulus (*G*′), as a function of hydration time, for cement pastes containing different amounts of MO with different SSA: (a) MO A, 220 m^2^·g^–1^, (b) MO B, 176 m^2^·g^–1^, (c) MO C, 98 m^2^·g^–1^, and (d) MO–D, 53 m^2^·g^–1^. The dashed vertical lines at 30, 60, and 240 min indicate the times used for obtaining the response surfaces.

Oscillatory tests performed in the linear viscoelastic domain were used to investigate the effect of the content of MO additives with different SSA on the kinetics of cement paste consolidation.^54,55^Figure 6(a-d) shows the evolution of the storage modulus (*G*′) of the paste as a function of time. For all the samples, regardless of the SSA or quantity of the MO additive, the storage modulus started higher than that of the pristine cement paste, with significantly higher *G*′ values during the first 20 min of the oscillatory test. After this initial period, until around 90 min, the *G*′ values were statistically similar, indicating that the MO amount and SSA had no significant effects during this hydration period. However, at 240 min, the *G*′ values increased and remained higher than for the pristine cement paste reference. This effect was most notable for the samples containing a greater amount (3 wt %) of higher SSA MO (Figure 6(a)), where *G*′ increased 2.4-fold, compared to the pristine cement paste. This change of cement paste consolidation caused by the addition of high SSA MO was a further indication that regeneration of the LDH nanostructure altered the formation of cement hydration products by heterogeneous nucleation of the cement phases such as ettringite (AFt) and calcium monosulfoaluminate (AFm),^20,25,56,57^ in agreement with the X-ray diffraction results for the MO-enriched samples (discussed below).

**Figure 7:**
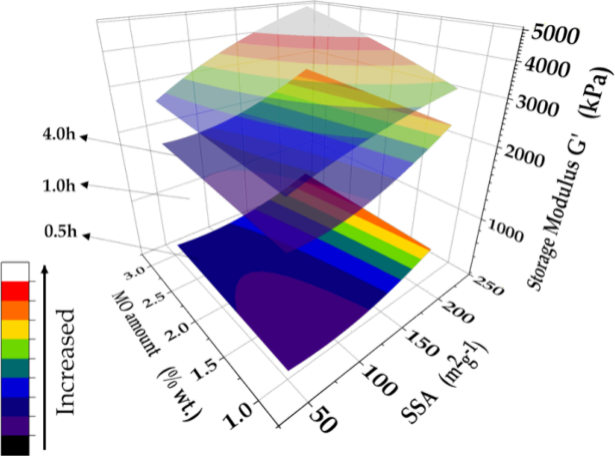
Response surfaces for storage modulus (*G*′) after 30, 60, and 240 min of hydration, as a function of the amount and SSA of MO added to the cement paste.

The hardening kinetics behaviors of the cement pastes are shown in the response surfaces obtained for the storage modulus (Figure 7), considering the different hydration periods of 30, 60, and 240 min, described by eqs 7, 8, and 9, respectively:
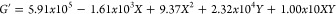
7
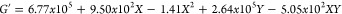
8
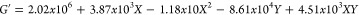
9where, X and Y are the SSA and amount of MO added to the cement paste, respectively.

Comparison of the XY interaction parameter coefficients of the equations showed that the hydration time influenced the response surface profile, which was almost flat for the shortest time, concave for 60 min, and slightly convex for 240 min. Both the SSA and amount of MO directly influenced the paste hardening kinetics. Irrespective of the time and SSA value, the paste storage modulus invariably increased with the amount of MO additive. An increase of the *G*′ value was observed when the MO SSA was higher than 98 m^2^·g^–1^. However, for SSA lower than 98 m^2^·g^–1^, a decrease of the *G*′ value was expected during the first 30 min of hydration, as indicated by the negative slope coefficient of the X variable in eq 7. This feature suggested that increase of the MO SSA favored the heterogeneous nucleation, accelerating the cement paste hydration and consolidation reactions. This acceleration of the storage modulus highlights the potential of these MO-modified pastes for different applications, such as 3D printing of cementitious materials, since they can comply with the main processing requirements, including (i) adequate initial fluidity for shaping by pumping or extrusion, and (ii) accelerated paste consolidation, avoiding flow/deformation under the influence of gravity.^20,58−60^

### 3.3 Effect of MO Additive on Hydrated Cement Structure

#### 3.3.1 Porous Texture

**Figure 8:**
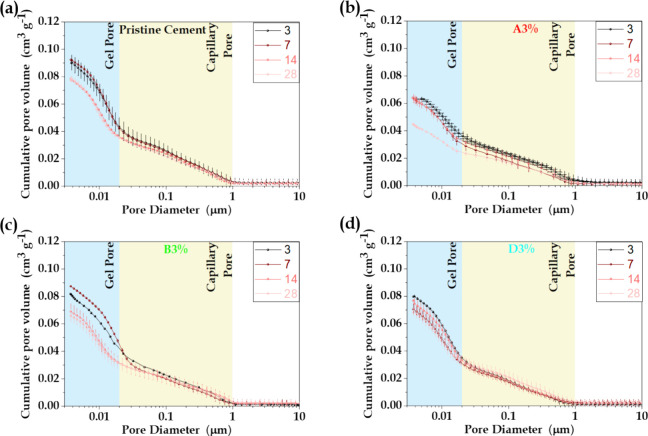
Effect of hydration time on the cumulative pore size distributions of the (a) pristine cement paste and the paste with addition of 3 wt % MO; (b) A3%, (c) B3%, and (d) D3%.

The porous texture is an important determinant of the mechanical properties and durability of cementitious materials.^61,62^ The porosity of cement samples at different hydration ages (3, 7, 14, and 28 days) was analyzed by mercury intrusion porosimetry (MIP). Figure 8(a–d) shows the cumulative intrusion pore size distribution curves for samples of pristine cement paste and the paste with 3 wt % of MO additive (samples A3%, B3% and D3%). The pore size distributions of all the samples were bimodal, presenting the typical families of gel pores (<20 nm) and capillary pores (50–2000 nm).^63−65^ A more notable measured porosity reduction of 36.9% was observed for sample A3% with MO additive.

**Figure 9:**
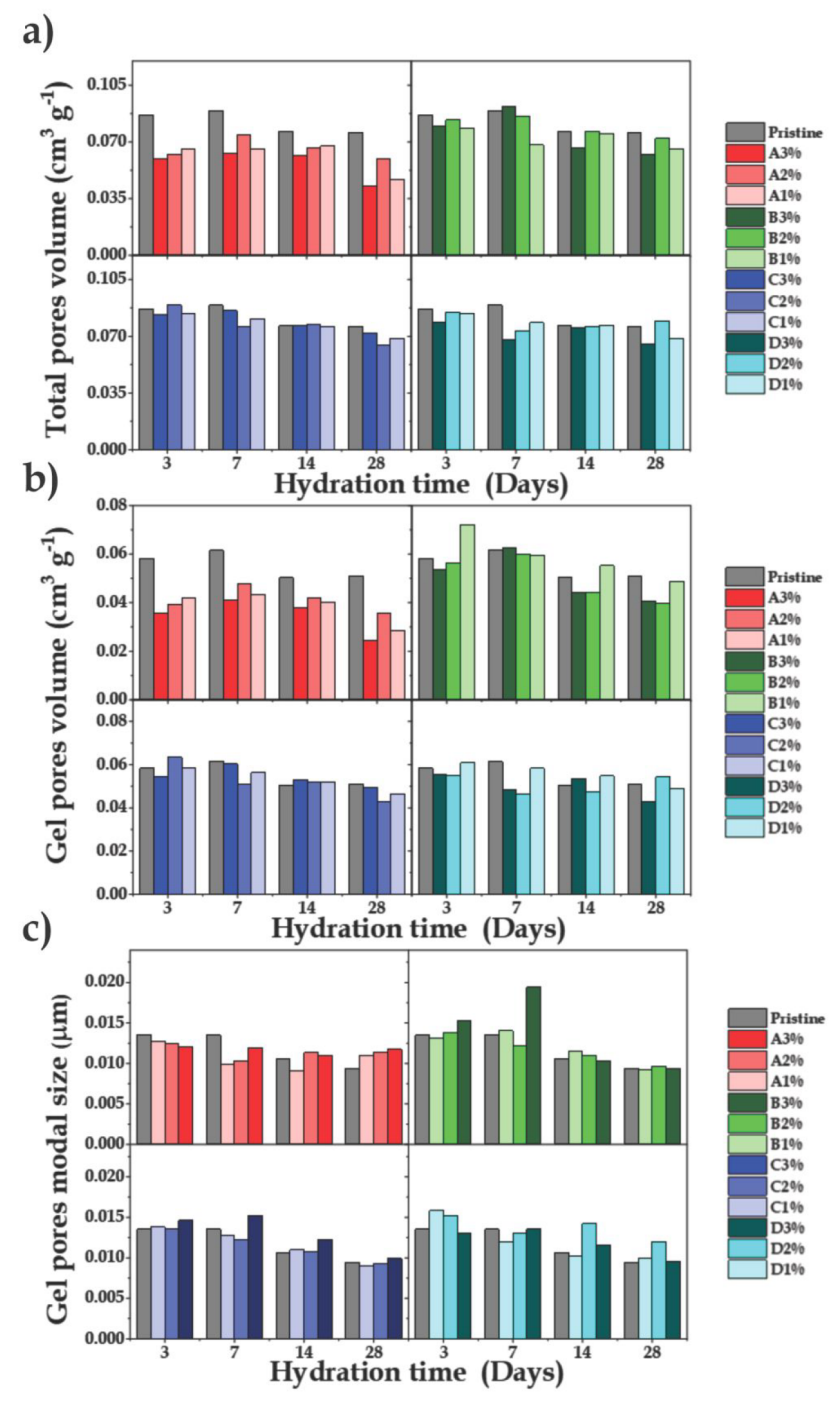
(a) Total pore volume, (b) gel pore volume, and (c) modal gel pore size, as a function of hydration time (3, 7, 14, and 28 days), for cements prepared with different amounts of different MO.

The total pore volume and the gel pore volume for all the samples and the different aging periods were used in Figures 9(a - c) to highlight the effects of MO amount and SSA on the pore texture of the aged cement. Higher SSA led to a greater reduction of the total pore volume, while the similar evolution observed for the gel pore volumes provided clear evidence that the capillary pore volume was almost invariant.

In addition, a trend of decreasing modal pore size with aging time was observed for most of the samples. The greater pore volume reduction for sample A3% indicated that the MO-LDH memory effect was able to eliminate the pores in the fresh cement. This sample also showed a substantial supplementary reduction of the pore volume after 28 days, indicating that the MO additive also modified the hydration kinetics of the paste. This behavior was consistent with recent work by Araujo et al., who reported crystallization of the LDH phase inside the pores of the cementitious matrix.^25^

**Figure 10:**
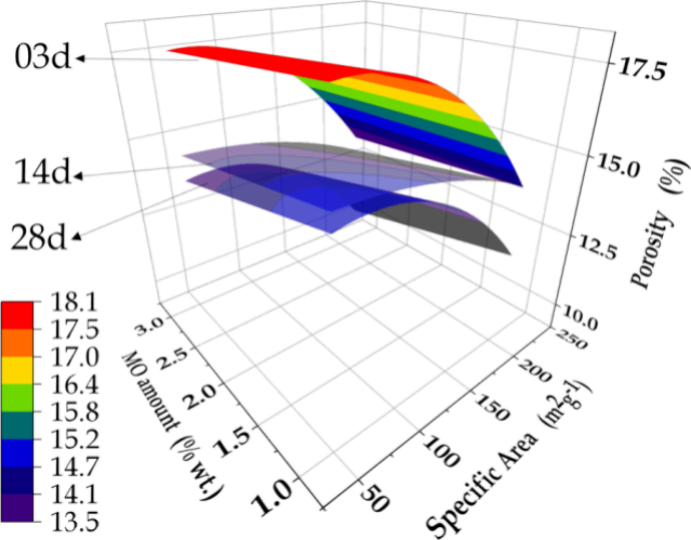
Dependence of porosity on the quantity and SSA of MO additive in the cement hydrated during 3, 14, and 28 days.

The dependence of porosity on the SSA and amount of MO added to the samples is shown in the response surfaces (Figure 10) corresponding to different hydration times. The profiles showed linear and parabolic relations between porosity and the MO amount and SSA, respectively, as indicated in eqs 10, 11, and 12, describing the evolution of the porosity (P) of the cement hydrated during 3, 14, and 28 days, respectively.
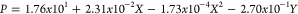
10
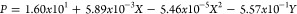
11
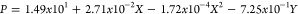
12where, X and Y are the SSA and amount of MO added to the cement, respectively.

The decrease of porosity over the hydration time can be seen from the vertical downshift of the response surfaces. The contributions of the SSA and amount of MO showed complementary behavior, as observed previously, with the second order term related to SSA indicating the existence of a maximum porosity for an SSA value given by the first derivative of eqs 10, 11, and 12. As expected, the lowest porosity was found for the sample containing the MO additive with the highest SSA, irrespective of the hydration time. A point worth noting is that the response surfaces for the cement aged during 14 days showed much less pronounced variations in porosity, as a function of MO SSA, compared to the profiles for the cement samples aged during 3 and 28 days. This could be explained by water competition between the MO-LDH memory effect and the cement gel phases present during the paste hydration stage. Higher SSA favored the consumption of water, leading to a reduction of the hydrated cement phase responsible for the formation of gel porosity. In addition, the increase of SSA enhanced the MO reactivity, favoring heterogeneous nucleation of the cement phase, with consequent water swelling of the C–S–H gel and the growth of LDH inside the gel pores, both of which contributed to reducing the porosity.

The observed contribution of the LDH memory effect to the reduction of the hydrated cement porosity could clearly play an important role in obtaining high-strength concrete, due to the greater number of particles in contact and improved stress distribution. Porosity is also directly linked to the leaching of constituents from cement bodies. Smaller or less connected pores lead to lower effective diffusivity, making the matrix less susceptible to degradation caused by leaching, consequently increasing the durability of cementitious materials.^66^

#### 3.3.2 Hydration Phases

**Figure 11:**
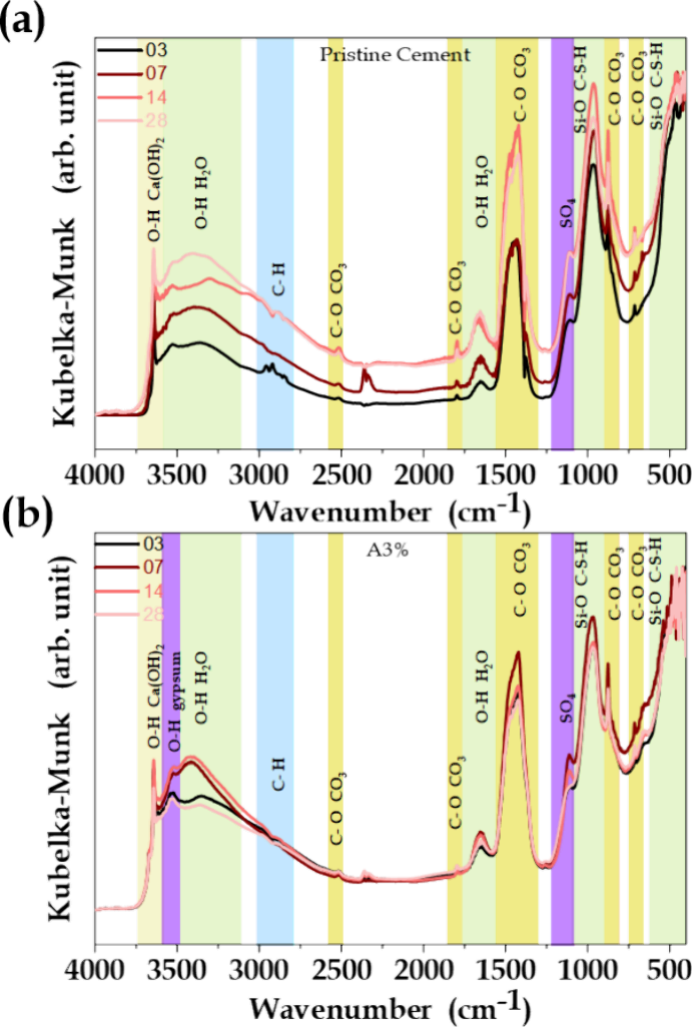
FTIR spectra of (a) the pristine cement and (b) the A3% sample after 3, 7, 14, and 28 days of hydration.

The rheological behavior results and the changes in the porous structure of the hydrated cement suggested that the incorporation of reactive particles with high specific surface area affected formation of the cement hydration phases. Fourier transform infrared spectroscopy (FTIR) was used to study the transformations of the pristine cement and A3% samples over different hydration periods (Figure 11). The bands for which the intensity increased with hydration time included a band at 3644 cm^–1^, attributed to the O–H bonds in the structure of portlandite (Ca(OH)_2_), as well as bands at around 3374 cm^–1^, assigned to O–H stretching of basanite and syngenite.^67^ Bands attributed to different carbonate vibrational modes also became more intense with increasing hydration time.

Although the FTIR profiles of the samples were similar, the spectra for the cements with addition of MO showed some clear differences, compared to the pristine cement, irrespective of the amount of additive and the aging time (Figure S4). For the samples with additive, the bands attributed to carbonate vibrations were less intense and the intensities did not increase significantly with hydration time. This evidenced that the additives modified the reaction kinetics of the cementitious material, as can be seen from the evolution of bands in the region between 3526 and 3100 cm^–1^, as the hydration time increased. In the case of the pristine cement, the bands in this region only became more intense with time, while this behavior was clearly modified for the samples with additives. After 7 days of aging, an intense signal at 3403 cm^–1^ could be attributed to the O–H vibrations of portlandite and water molecules confined in capillary structures.^67,68^ These features supported the ability of the additive to act in the gel pore and mesopore regions, hindering the cement carbonation reactions.

**Figure 12:**
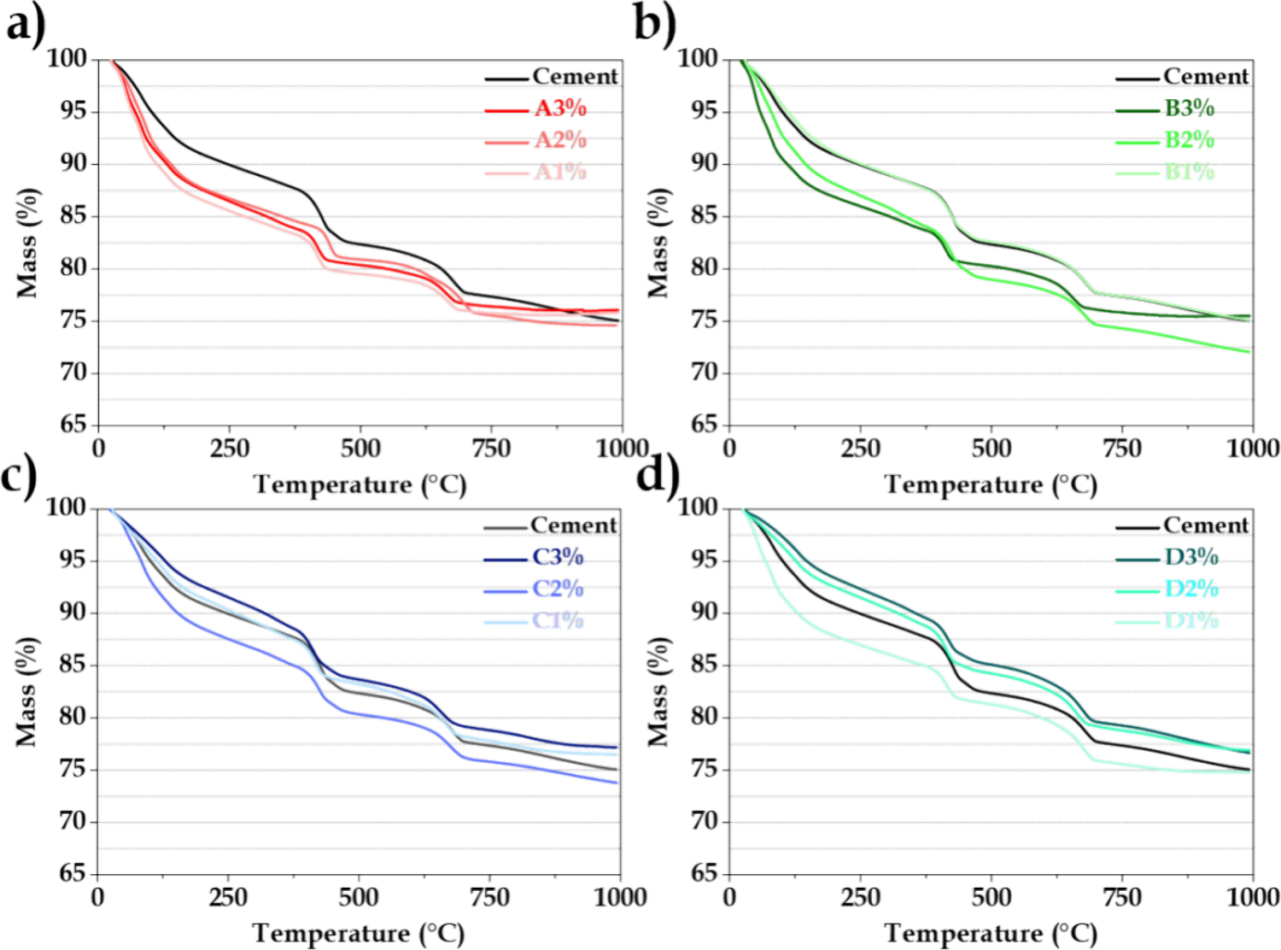
TGA curves for the cement samples hydrated for 3 days, as a function of the quantity of different MO: (a) pristine cement, A3%, A2%, and A1%, (b) pristine cement, B3%, B2%, and B1%, (c) pristine cement, C3%, C2%, and C1%, and (d) pristine cement, D3%, D2%, and D1%.

Figure 12 shows the TGA curves for the pristine and modified cement samples after 3 days of hydration. The main thermal decomposition steps were indicated by three sequential mass loss events. The first event, between 25 and 225 °C, was attributed to dehydration, mainly related to water losses from the hydrated structures of AFt/AFm and C–S–H gel.^69,70^ The second event, between 310 and 510 °C, could be mainly explained by the decomposition of Ca(OH)_2_. The third thermal event, in the region between 520 and 780 °C, was attributed to decarbonation of the CaCO_3_ composite.^69^Figure S5 presents the mass loss percentages related to the first event for each sample prepared with different hydration periods. The samples containing MO presented larger mass losses during the earlier hydration period, whereas the pristine cement exhibited an approximately invariant mass loss, clearly indicating that the materials with MO contained more water at 3 and 14 days of hydration. This confirmed the ability of the MO additive to increase the amount of confined water, which could be explained by the presence of a greater quantity of hydrated phases such as AFt/AFm and C–S–H gel. However, after 28 days, the mass loss associated with this event was approximately the same for both the pristine cement and the modified cement samples.

Figure S6 shows the mass losses for the second event, corresponding to the decomposition of Ca(OH)_2_. For hydration periods of 3 and 7 days, the samples containing MO showed lower mass losses, compared to the pristine cement. However, after 14 and 28 days, the mass losses were similar for all the samples. This behavior suggested that the presence of the additives delayed the formation of portlandite. For the third thermal event (Figure S7), related to sample decarbonation, the mass losses were approximately constant between 3 and 14 days of hydration, while after 28 days, the pristine cement showed an increase of mass loss, different to the samples containing MO. These findings demonstrated a significant influence of the MO additive on the cement hydration kinetics, with an increased water content and reduced presence of portlandite and initial carbonate. These changes, together with the previously observed increase of the elastic storage modulus and reduced porosity, could be attributed to hydration competition between the cement and MO. These findings emphasized by the first time the substantial impact of the MO memory effect, dependent on MO SSA and quantity, which markedly altered the properties of the modified cement.

**Figure 13:**
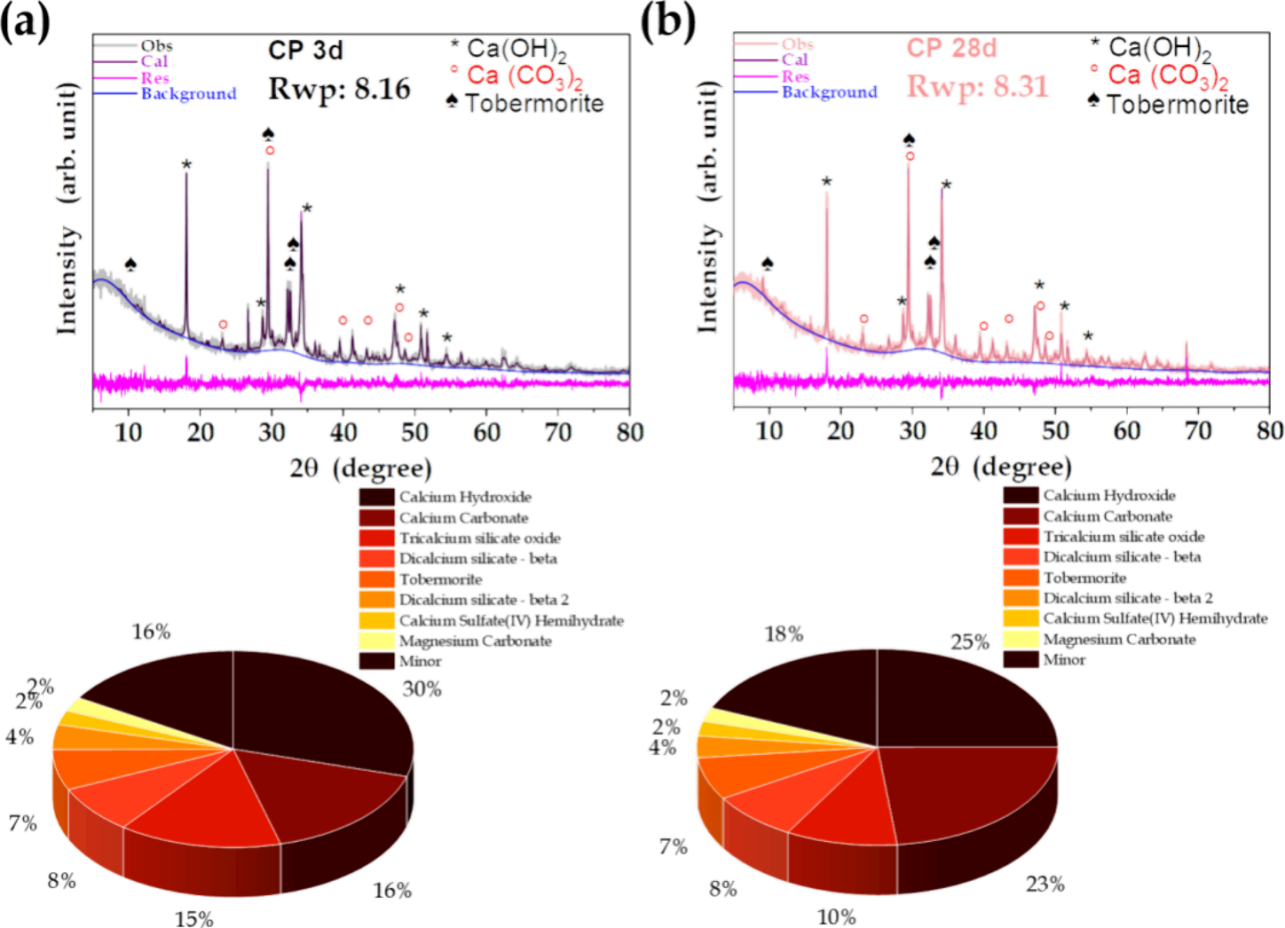
X-ray diffractograms of the pristine cement and quantification of the crystalline phases observed after (a) 3 and (b) 28 days of hydration.

Figure 13 shows X-ray diffractograms of the pristine cement hydrated during 3 and 28 days. After hydration, the cement formed new crystalline phases consisting predominantly of Ca(OH)_2_ and CaCO_3_. The hydration of the C3S phases occurred rapidly after the first contact with water, leading to the formation of calcium hydroxide and C–S–H gel (eq 13). The hydration process of dicalcium silicate (eq 14) is analogous to that of C3S, but slower. In the presence of calcium sulfate, tricalcium aluminate (3CaO·Al_2_O_3_ or C_3_A) hydrates and forms ettringite (Ca_6_A_l2_(SO_4_)_3_(OH)_12_·26H_2_O, AFt or trisulfate), as shown in eq 15.
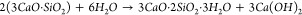
13
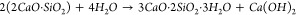
14
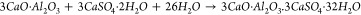
15

The diffractograms for the pristine cement showed slight alterations, as a function of hydration time, including the appearance of a diffraction peak at 9° after 14 and 28 days, characteristic of the AFt phase. Cement carbonation is a natural process that can proceed according to various chemical pathways. In a CO_2(g)_-rich atmosphere, there is a potential decrease of pH as carbon dioxide gas dissolves in pore water and hydrates to form H_2_CO_3_. This, in turn, rapidly ionizes to produce HCO_3_^–^ and CO_3_^2–^, depending on the pH level. The presence of these species may lead to the formation of calcium carbonate (eqs 16-21).^71^ Analysis of the pristine cement samples revealed key structural changes over time, notably a decrease of Ca(OH)_2_ and an increase of carbonation. These changes significantly affected the concentrations of hydration products (C–S–H gel, calcium hydroxide, and ettringite) at the end of the initial hydration period.
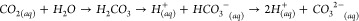
16
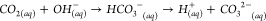
17
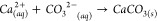
18
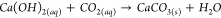
19
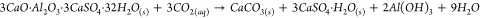
20
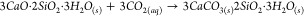
21

For the cement samples containing MO, distinct changes occurred within 3 days of aging, indicating an alteration in the kinetics of cement consolidation. Specifically, there was increased formation of the ettringite phase, suggesting enhanced reactivity due to the additives.

The hydration process of C3S (eq 13) primarily involves dissolution and precipitation, influenced by both under-saturation and oversaturation conditions. During hydration of the cement containing MO, the competition for water between the cement and MO altered the balance of available water. This change affected the dissolution of C3S, consequently affecting the hydration kinetics and leading to observed modification of the hydraulic properties of the paste and the porosity of the final composite.

**Figure 14:**
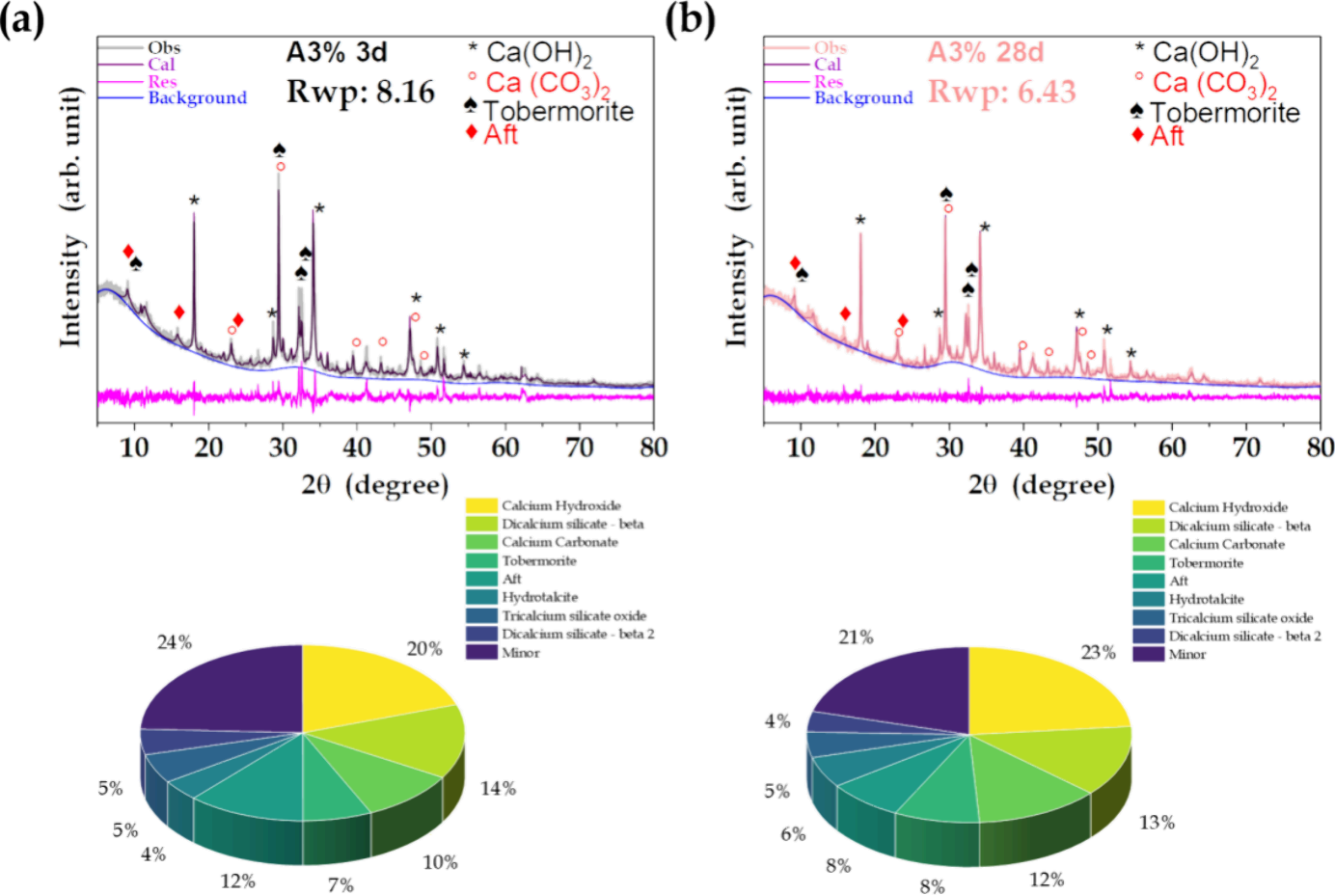
X-ray diffractograms of the A3% samples containing MO and quantification of the crystalline phases observed after (a) 3 and (b) 28 days of hydration.

Dissolution and precipitation processes are closely linked, while the formation of portlandite crystals is restricted by the presence of silicate ions. Recent findings suggest that C–S–H and portlandite might both originate from the same initial points.^72^ The dynamic interaction nature of dissolution, saturation, nucleation, and growth in cement chemistry is demonstrated by the opposing effects of different quantities of Ca(OH)_2_. Small amounts slow down the hydration process by altering saturation levels, whereas the introduction of more significant quantities of portlandite reduces this delay, due to the increased availability of nucleation sites. For the A3% cement, a notable reduction (about 10%, after 3 days) of the initial Ca(OH)_2_ content was observed, together with substantial increases in the amounts of AFt and hydrotalcite phases, with the peak at 9° appearing within 3 days of hydration of the cement containing MO (Figure 14). This finding indicated that the MO additive not only accelerated the crystallization of these cement hydration phases, but also significantly affected the initial calcium hydroxide levels. Regardless of the hydration duration, the pristine cement exhibited a carbonation effect, evidenced by an approximately 5% decrease of the Ca(OH)_2_ phase and a 7% increase of the CaCO_3_ phase. Conversely, the A3% sample showed a 3% increase of Ca(OH)_2_ and only a 1.6% increase of CaCO_3_. A significant reduction was observed in the quantity of carbonates, of up to 12% for the A3% sample after 28 days of hydration, compared to the pristine cement.

The XRD results corroborated the FTIR and TGA observations, confirming the alteration in hydration kinetics induced by the presence of the MO additive. These results further emphasized the significant impact of the MO additive on the cement hydration kinetics. In summary, this impact was characterized by increased water content, decreases of portlandite and initial carbonate, and improvement of both the elastic storage modulus and porosity. However, the mechanisms involved in formation of the cement hydration products are complex, since the processes can act in series, in parallel, or as a combination of the two, with the product of one step acting as a watering agent in the next one. Therefore, further studies are needed to obtain a deeper understanding of the effect of MO incorporation on cement chemistry.^73,74^

## 4 Conclusions

LDH materials with specific surface areas (SSA) controlled from 88 to 5 m^2^·g^–1^ were successfully prepared by increasing the isothermal aging time of LDH precipitated in aqueous solution from 0 to 30 h. This reduction of SSA was a consequence of increased crystallite size resulting from the growth of lamellae and aggregative stacking of layers.

The thermal decomposition of LDH enabled the preparation of mixed oxide (MO) with SSA controlled from 220 to 54 m^2^·g^–1^.

The memory effect involving regeneration of the LDH structure by the rehydration of MO constitutes a versatile strategy for improving the rheology of cement paste and the porosity of cementitious materials, by simple selection of the SSA and amount of the MO additive.

Irrespective of the SSA, the addition of a small amount (1%) of MO influenced the fluidity of the cement paste, due to a lubricating effect caused by the presence of delaminated LDH platelets. The addition of higher MO amounts (2 and 3%) increased the shear stress of the cement paste, due to water adsorption and growth of agglomerates.

Increase of the quantity and/or SSA of the MO additive significantly reduced the paste consolidation time and altered the hydration reactions, leading to decreased cement porosity. Structural characterizations confirmed the changes in the relative quantities of crystalline cement phases associated with the earlier formation of hydrated phases and reduced carbonation, influenced by the LDH memory effect.

The LDH memory effect influenced the cement hydration kinetics by modifying the water-cement balance, leading to reduced portlandite formation, accelerated formation of other hydrated phases, and decreased carbonation.

Overall, this study highlights the potential of MO-LDH additives for enhancing the properties of cementitious materials, such as improved rheology for 3D construction printing, as well as greater durability resulting from reduced porosity and scavenging of anions considered harmful to the metallic structure of reinforced concrete. The correlation between the properties of LDH and these structural changes in cement has been little explored in the literature, which demonstrates the relevance of the contributions of this work in areas such as nanotechnology in civil materials construction.

## Supporting Information Available

Compositions of the simulated cement pore solutions; SEM images of LDH A and LDH D additives; dynamic yield stress and plastic viscosity as a function of the specific surface and amount of MO additive in the cement paste; FTIR spectra for cement samples containing different amounts of MO; TGA mass losses (first, second, and third events) of the cement samples containing different amounts of MO, aged for 3, 7, 14, and 28 days (PDF)
