# Supplementary material for: Relevance of Specific Surface of Mixed Oxide Derived from Layered Double Hydroxides on the Rheological Properties and Porosity of Cement Pastes
Source: ACS Appl Mater Interfaces. 2024 Jun 5;17(9):13050–64. doi: 10.1021/acsami.4c01949 (PMC11891860; doi:10.1021/acsami.4c01949)
Supplement: Supplementary file 1 — am4c01949_si_001.pdf [file am4c01949_si_001.pdf]

Supporting Information

Relevance of Specific Surface of Mixed Oxide Derived from Layered Double Hydroxides on the Rheological Properties and Porosity of Cement Pastes

Caio C. dos Santos[a]*, Adriana A. Almeida[a], Sandra H. Pulcinelli[a], Celso V. Santilli[a]*

[a]Chemistry Institute of the São Paulo State University (UNESP), Araraquara, 14800-900, SP, Brazil.

*E-mail: caio.c.santos@unesp.br (C.C. dos Santos); cv.santilli@unesp.br (C. V. Santilli).

Supporting Tables

Table S1. Compositions of the simulated cement pore solutions (SCPS1 and SCPS2).

SCPS1		SCPS2	
Reagent	Concentration (mol L^{-1})	Reagent	Concentration (mol L^{-1})
KOH	0.30	$CaCO_3$	0.001
NaOH	0.10	Na_2SO_4	0.002
$Ca(OH)_2$	0.01	NaCl	0.0014
Na_2SO_4	0.002		
pH = 14		pH = 8	

Supporting Figures

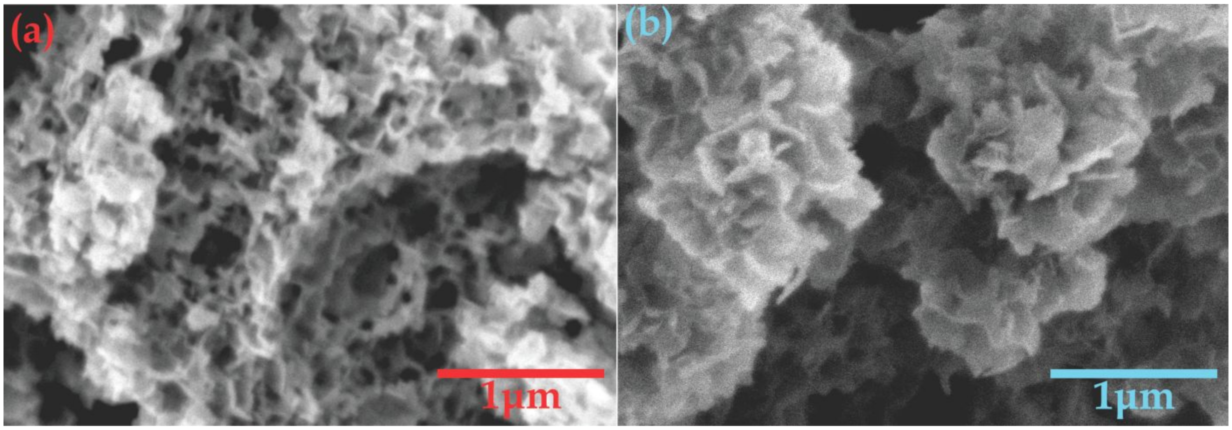

Figure S1. Scanning electron microscopy images of (a) LDH A and (b) LDH D additives.

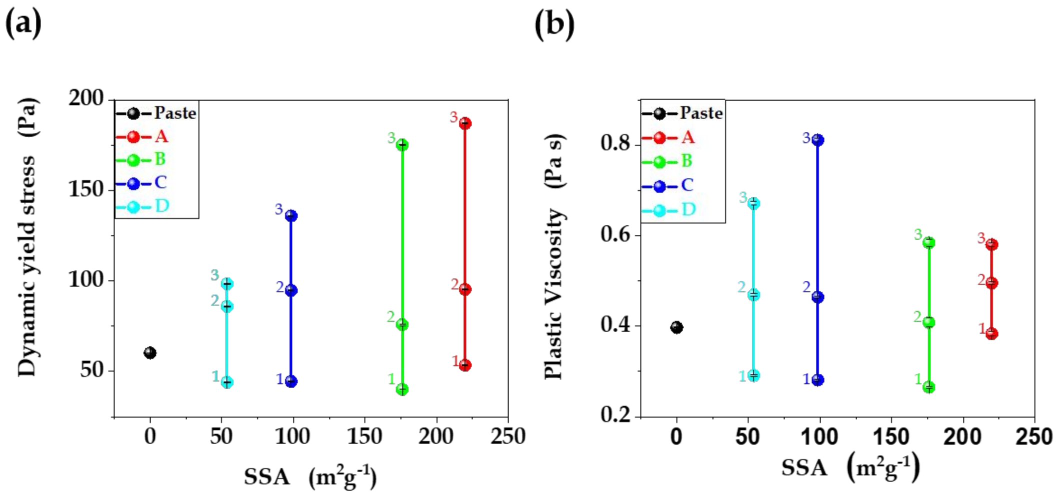

Figure S2. (a) Dynamic yield stress and (b) plastic viscosity, as a function of specific surface area of MO in cement pastes with different additive quantities (0, 1, 2, and 3 wt.%).

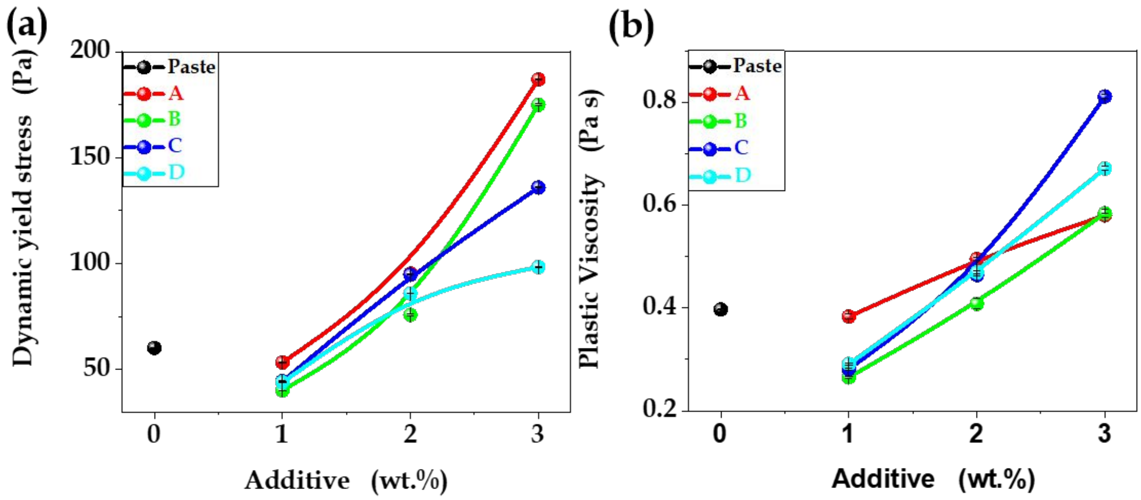

Figure S3. (a) Dynamic yield stress and (b) plastic viscosity, as a function of the amount of MO additive in the cement paste.

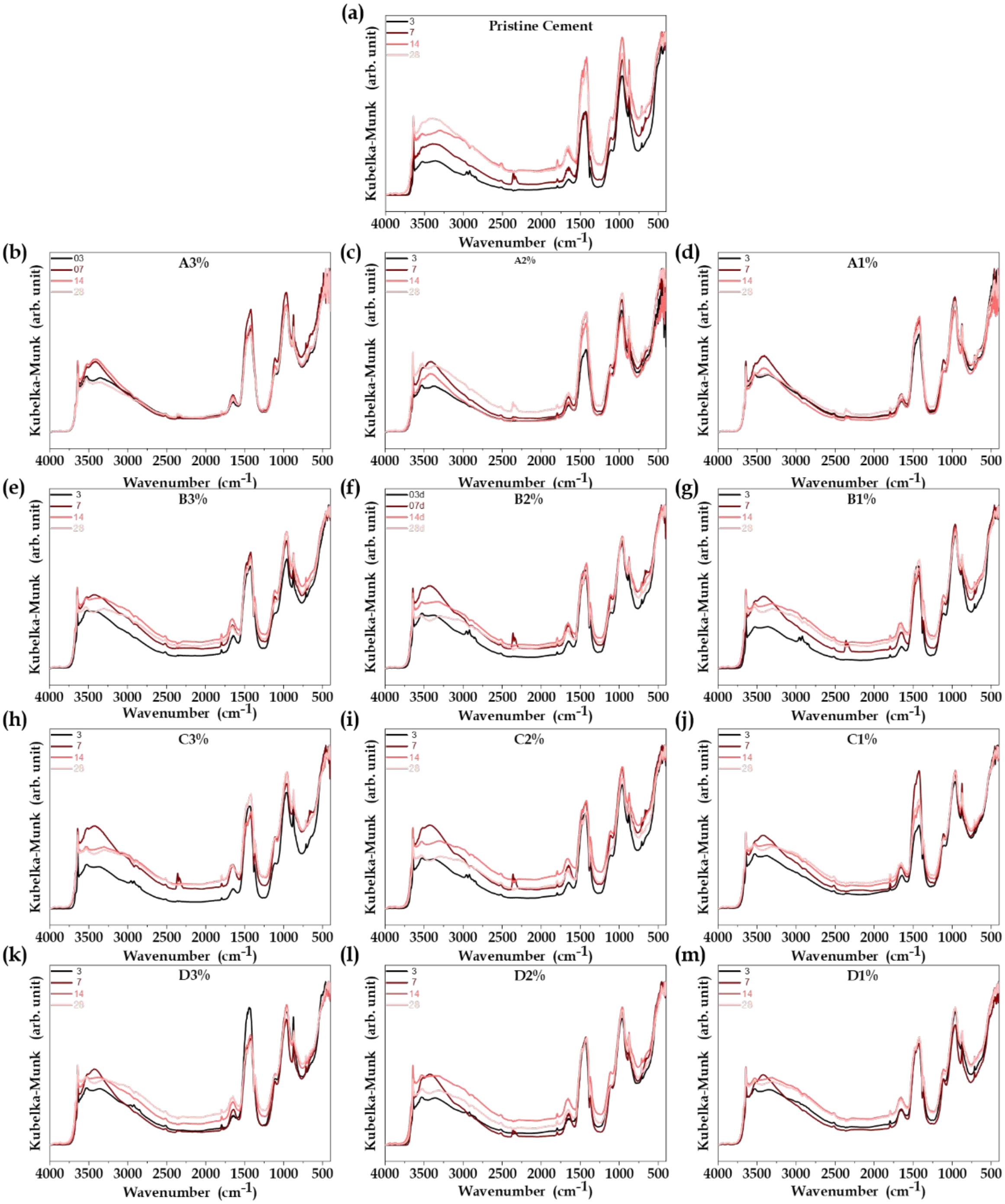

Figure S4. FTIR spectra of the pristine cement and the cement samples containing different amounts of MO, aged for 3, 7, 14, and 28 days. (a) Pristine cement, (b) A3%, (c) A2%, (d) A1%, (e) B3%, (f) B2%, (g) B1%, (h) C3%, (i) C2%, (j) C1%, (k) D3%, (l) D2%, (m) D1%.

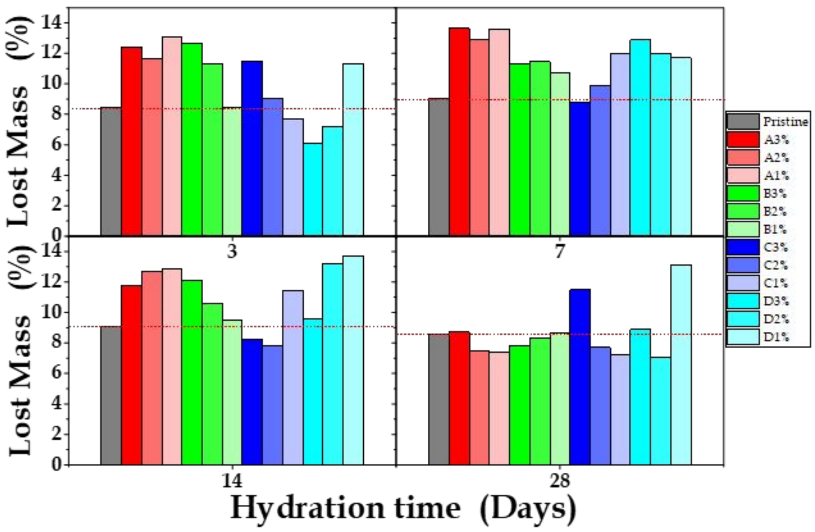

Figure S5. TGA mass losses (first event) of the pristine cement and the cement samples containing different amounts of MO, aged for 3, 7, 14, and 28 days.

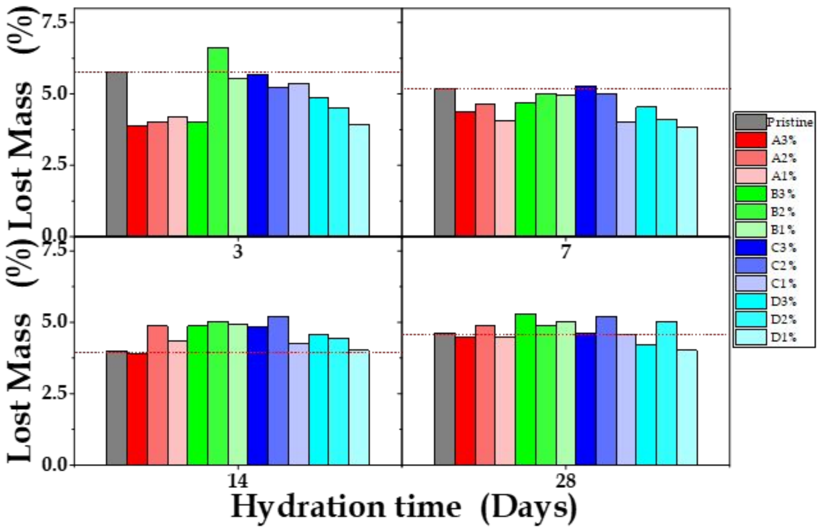

Figure S6. TGA mass losses (second event) of the pristine cement and the cement samples containing different amounts of MO, aged for 3, 7, 14, and 28 days.

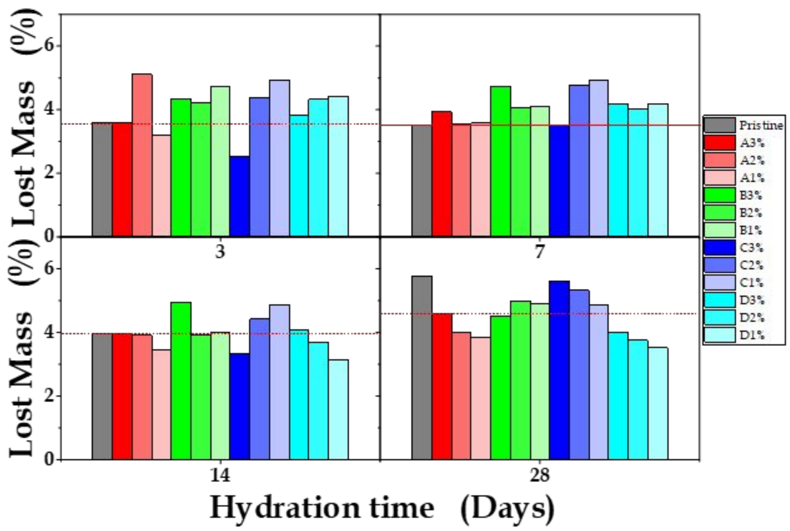

Figure S7. TGA mass losses (third event) of the pristine cement and the cement samples containing different amounts of MO, aged for 3, 7, 14, and 28 days.
